# Host genetic determinants of resistance and susceptibility to bovine leukemia virus infection: A mini-review

**DOI:** 10.1007/s11033-025-11230-7

**Published:** 2025-11-17

**Authors:** Mohammad Mehdi Akbarin, Zahra Farjami, Hugo Ramírez Álvarez

**Affiliations:** 1 Virology, Genetics, and Molecular Biology Laboratory, Faculty of Higher Studies Cuautitlan, Veterinary Medicine, Campus 4, National Autonomous University of Mexico. Cuautitlan Izcalli, Mexico, National Autonomous University of Mexico (UNAM), 54740 Cuautitlán Izcalli, Mexico; 2 Mashhad Medical Sciences-Medical School, Islamic Azad University, Mashhad, Iran; 3 Department of Biology, Islamic Azad University, Damghan, Iran; 4 Universidad Nacional Autónoma de México. FES-Cuautitlán, Carretera Cuautitlán-Teoloyucan Km. 2.5, San Sebastián Xhala, Cuautitlán Izcalli, 54714 Mexico

**Keywords:** BLV, SNP, BoLA-DRB3, TNF, GWAS, Genetic resistance

## Abstract

Bovine leukemia virus (BLV), a member of the *Deltaretrovirus* genus, causes enzootic bovine leukosis, leading to clinical outcomes that range from asymptomatic infection to malignant lymphoma. Host genetic factors significantly influence BLV susceptibility, proviral load (PVL), immune response, and disease progression. This mini-review synthesizes evidence on genetic polymorphisms in immune-related genes such as *BoLA-DRB3*, *Tumor necrosis factor (TNF)*, and *immunoglobulin loci*, and examines novel findings from genome-wide association studies (GWAS). Beyond classical immune genes, recent GWAS have identified novel loci including *SPATA16 (spermatogenesis associated 16)*, *ABT1 (activator of basal transcription 1)*, *IER3 (immediate early response 3)*, *Adaptor Related Protein Complex 4 Subunit Beta 1 (AP4B1)*,* Tripartite Motif Containing 45 (TRIM45)*,* Patatin Like Phospholipase Domain Containing 1* (*PNPLA1)*, and *PRRC2A (proline-rich coiled-coil 2 A)* that are implicated in transcriptional regulation, stress response, RNA processing, and intracellular transport, all of which may modulate viral replication and persistence. Understanding these genetic determinants provides new insights into host-virus interactions and offers opportunities for selective breeding strategies, biomarker development, and improved BLV control programs.

## Introduction

 Bovine leukemia virus (BLV), a member of the *Deltaretrovirus* genus, is the causative agent of enzootic bovine leukosis, a chronic lymphoproliferative disease in cattle [1, 2]. The virus primarily targets B lymphocytes to initiate infection, then promoting their clonal expansion and, in some cases, progressing to lymphoma [2]. The wide range of clinical outcomes from asymptomatic carriers as primary outcome to animals with overt leukemia suggests that host genetics significantly influences the progression and immune response to BLV infection [3, 4].

In recent years, increasing attention has been given to single-nucleotide polymorphisms (SNPs) in the host genome and their potential role in modulating susceptibility and disease severity in BLV-infected cattle [2, 5]. As the most prevalent form of genetic variation, SNPs are known to affect immune responses, virus-host interactions, and disease risk [5, 6]. In the context of BLV, specific SNPs in immune-regulating genes including those in the *Major Histocompatibility Complex* (*MHC*), cytokine pathways, and immune checkpoint molecules can influence the host’s ability to detect and eliminate the virus [7–9].

Among these genetic factors, variation within the bovine MHC, known as the *BoLA* region, is significant [10]. Specific *BoLA* alleles have been linked to either resistance or increased vulnerability to BLV infection and the development of lymphoma [11]. Recognizing and understanding this genetic diversity is essential for breeding programs that aim to enhance resistance, improve herd health, and reduce economic losses. Moreover, knowledge of host genomic factors could inform future development of targeted vaccines and immunotherapies.

Genes encoding interleukins, interferons, and other cytokines are also subject to SNP-related changes, which can influence the effectiveness of antiviral responses. For example, promoter polymorphisms in the *TNF-α* gene have been linked to altered immune activation and BLV susceptibility [12]. Comparative analyses in water buffaloes further demonstrated breed-dependent variation in cytokine responses, including IL-4, IL-6, IL-10, IL-12p40, and IFN-γ expression during BLV infection [13]. Additionally, genetic variation in the *TNF receptor II* gene has been associated with differential TNF-mediated immune responses in BLV-infected cattle [14].

This mini-review specifically focuses on single-nucleotide polymorphisms (SNPs) and other host genetic variations that influence BLV susceptibility, Proviral load (PVL) as a key quantitative measure of BLV infection burden, and disease progression, while excluding non-genetic or purely mechanistic aspects to maintain a concise and consistent scope. We highlight key immune-related loci and their impact on infection outcomes, providing a molecular framework for host-virus interactions and exploring opportunities for genetically based disease mitigation strategies in cattle.

## The correlation of host genome variations and BLV infection

### Major histocompatibility complex

Genetic variation within the *MHC* plays a crucial role in shaping the host immune response to BLV (Table 1). The bovine *MHC* region, particularly the Bovine Leukocyte Antigen (BoLA) complex, is central to antigen presentation and significantly influences the host’s ability to control viral replication [15].

**Table 1:** Summary of key findings from MHC-SNP study and BLV infection

Study (Year) Ref #	Sample Number	Breed	Study design	PVL assay/threshold	Main endpoint	Effect direction & size	*p*-value/Odds Ratio
Takeshima et al. (2017) [8]	676	Japanese Black	GWAS; stratified by PVL (cross-sectional)	qPCR; high vs. low PVL: 1 copy/10^5^ cell Highest PVL: 132,230 copy/10^5^ cell	SNPs (2 in *BoLA* region, 1 near CNTN3) associated with PVL	*BoLA*-linked SNPs → lower PVL	**rs29026690** on BTA23: *p* = 1.91 × 10⁻⁷ (odds ratio [OR] = 2.745) **rs17872126** on BTA23: *p* = 1.91 × 10⁻⁷ (OR = 0.414) **rs110616206** on BTA22 (near CNTN3): *p* = 5.37 × 10⁻⁷ (OR = 6.589)
Zhou et al. (2007) [9]	348	Mixed New Zealand cattle	PCR-SSCP genotyping (allelic diversity)	NA	*BoLA*-*DRA* allelic variants (synonymous) identified	Diversity described; no direct PVL endpoint	NA
Notsu et al. (2022) [16]	846	Japanese Black (2 prefectures)	Cross-sectional survey	qPCR; low/undetectable PVL (exact threshold NA)	Frequency and impact of *BoLA*-DRB3*009:02 on PVL	*BoLA*-DRB3*009:02 → resistant (3.8% overall)	*p* < 0.05
Hamada et al. (2023) [11]	107	Holstein (Egypt)	Cross-sectional association	qPCR; Low PVL (LPVL)34–85 Moderate PVL 89–25,858 High PVL (HPVL) 35,138–98,725 copies/10⁵ cell	BoLA-DRB3*010:01 alleles vs. PVL	BoLA-DRB3*010:01 → low PVL; BoLA-DRB3*015:01 → high PVL; BoLA-DRB3*012:01 → high PVL	*p* < 0.0001
Inenaga et al. (2023) [17]	1,810	Japanese Brown (Kumamoto), Japanese Black, Holstein	Comparative cross-sectional	PVL measured; Low PVL: <1,000 copies/µg DNA High PVL: 1000–15,000 copies/µg DNA	Breed differences in PVL and BLV positivity	Kumamoto strain → lower PVL; >80% <10,000 copies/µg	*BoLA*-*DRB3* allele effect on PVL, *P* = 0.08851 PVL (JBRK vs. JB & HF; all BLV-infected), p = *P* < 0.00005 PVL (59 JBRK vs. 111 JB; same farms), p = *P* < 0.001

NA: Not Applicable

SNP: Single Nucleotide Polymorphism

rs: Reference SNP

Recent studies have highlighted the relationship between *MHC* polymorphisms and variations in PVL and disease progression. For instance, a genome-wide association study (GWAS) conducted by Takeshima et al. (2017) involving 676 Japanese Black cattle (93 with high and 266 with low PVL) identified three SNPs significantly associated with BLV integration levels. Two of these SNPs were located within the *BoLA* region on chromosome 23, and one was near the *Contactin 3 (CNTN3)* gene on chromosome 22 [8]. These findings suggest that *MHC*-linked genetic variation, beyond the previously reported class II effects, contributes to controlling BLV PVL burden.

Another key allele, BoLA-DRB3*009:02, has been associated with resistance to BLV. Notsu et al. (2022) surveyed 846 Japanese Black cattle and found this allele present in 3.8% of the population, with a higher frequency in one prefecture (8.6%) than another (2.6%) [16]. Cattle carrying this allele generally had low or undetectable PVL, supporting its potential role as a resistance marker in breeding programs.

Further supporting the role of breed-specific genetic resistance, Inenaga et al. (2023) compared PVL levels among Japanese Brown (Kumamoto strain), Japanese Black, and Holstein-Friesian cattle. The Kumamoto strain exhibited notably lower PVL levels over 80% had PVL < 10,000 copies/µg DNA and fewer BLV-positive cases overall [17]. Importantly, no association was found between *BoLA-DRB3* haplotypes and PVL, indicating that non-*DRB3* genetic factors might underlie this resistance.

Similarly, Hamada et al. (2023) investigated *BoLA-DRB3* polymorphisms in 107 BLV-infected Holstein cattle in Egypt and identified 18 different alleles. Among these, BoLA-DRB3*010:01 was significantly associated with low PVL (suggesting resistance), whereas BoLA-DRB3*015:01 correlated with high PVL (suggesting susceptibility) [11]. Interestingly, BoLA-DRB3*012:01 which has shown protective effects in Japanese Black cattle was associated with high PVL in this Holstein cohort, illustrating how the impact of BoLA-DRB3*012:01 alleles may vary across breeds and geographic regions.

These findings underscore the crucial role of *MHC* diversity in BLV resistance, providing valuable insights into the key factors influencing disease resistance in cattle.

### Immune response genes

#### Immunoglobulin (Ig) genes

They are fundamental to the adaptive immune system, enabling the recognition and neutralization of a wide array of pathogens, including BLV. The diversity generated through the recombination of variable (V), diversity (D), and joining (J) gene segments allows for broad antibody specificity.

In the context of BLV infection, early work by Saini et al. (1997) examined the antibody repertoire in BLV-infected cattle and reported a restricted usage of the *Bov VH1* immunoglobulin heavy-chain variable region family [18]. All IgM-secreting hybridomas analyzed utilized the Bov VH1 family, which, despite being polymorphic, consistently featured extended complementarity-determining region 3 (CDR3) sequences (15–23 amino acids) [18]. These findings suggest a focused yet adaptable antibody response to BLV antigens. The limited diversity observed may reflect viral strategies to evade humoral immunity or inherent genetic constraints within the bovine immune repertoire.

However, to date, no studies have identified specific single-nucleotide polymorphisms (SNPs) within immunoglobulin genes that are associated with BLV susceptibility or disease progression. Most contemporary research has shifted toward immune-regulatory genes (e.g., *BoLA*, cytokine-related loci) and genome-wide association studies (GWAS) to identify novel markers of susceptibility and resistance. This lack of recent evidence highlights an important research gap, as understanding Ig gene polymorphism and expression could provide valuable insights into humoral immunity, antibody-mediated viral control, and vaccine design.

#### Tumor necrosis factor

Tumor necrosis factor (TNF) is a key pro-inflammatory cytokine that plays a central role in orchestrating immune responses during viral infections. Produced by activated macrophages, T cells, and other immune cells, TNF contributes to antiviral defense by promoting inflammation, triggering apoptosis in infected cells, and recruiting immune effectors [19, 20]. However, excessive TNF production can also lead to immunopathology, highlighting the delicate balance between viral control and immune-mediated tissue damage [21].

In 2006, Yudin et al. presented findings at a conference (Proceedings of the Fifth International Conference on Bioinformatics of Genome Regulation and Structure), identifying a promoter SNP (–824 A > G) in the *TNF-α* gene that was associated with altered immune profiles in infected cattle (supplementary reference). This observation aligns with subsequent peer-reviewed studies (Bojarojc-Nosowicz et al., 2015; Stachura et al., 2016; Ohnuki et al., 2021), which confirmed the role of *TNF-α* polymorphisms in modulating immune responses to BLV infection.

Building on these findings, Bojarojc-Nosowicz et al. (2015) reported that cattle with the G/G genotype had elevated levels of membrane-bound TNF-α (mTNF-α) on CD11b⁺ and IgM⁺ cells, despite showing reduced TNF-α mRNA expression overall [22]. This discrepancy suggests that complex post-transcriptional regulation, influenced by the host genotype, is involved during BLV infection.

Further evidence linking host genetics to disease progression came from Ohnuki et al. (2021), who developed a BLV-specific DNA-capture sequencing approach capable of analyzing the whole viral genome, integration sites, and associated host SNPs [23]. Their study confirmed the involvement of the *TNF-α*−824 G variant in viral integration and clonal expansion, reinforcing the gene’s role in modulating BLV-related immune dynamics.

Additionally, a study by Mingala et al. (2009) in Philippine water buffaloes found that BLV-infected animals exhibited upregulated expression of IL-4, IL-6, IL-10, and IL-12 p40, but no significant change in IFN-γ levels [13]. Comparative promoter analysis revealed that swamp-type buffaloes expressed higher levels of TNF-α and IFN-γ than riverine buffaloes, attributed to multiple SNPs in promoter regions. These findings suggest that breed or subspecies-specific promoter polymorphisms influence cytokine expression and may underlie differential disease tolerance.

Cheng et al. (2016) further demonstrated the broader relevance of *TNF-α* polymorphisms by linking promoter variants including one corresponding to rs1800630 to altered susceptibility to Mycobacterium bovis co-infection in cattle [24]. These variants likely modulate cytokine expression and immune regulation. Although this study did not directly focus on BLV, its findings are relevant because the same *TNF-α* promoter variants have been implicated in modulating immune responses during BLV infection. Both *M. bovis* and BLV are pathogens that exploit host immune regulation, and *TNF-α* plays a central role in orchestrating inflammation, macrophage activation, and antiviral defense. Therefore, the work of Cheng et al. provides comparative evidence that *TNF-α* genetic variation can influence susceptibility to multiple bovine infectious diseases, reinforcing its importance in shaping disease outcomes, including BLV infection.

Finally, Stachura et al. (2016) investigated the *TNF receptor II (TNF-RII)* gene in BLV-infected Polish dairy cattle. They focused on the rs42686850 SNP and a microsatellite repeat (CA)n in intron 1, both of which were associated with changes in *TNF-RII* mRNA expression and the proportions of TNF⁺ immune cell subsets [14]. These findings suggest that genetic variation in both TNF and its receptor contributes to shaping immune responses during BLV infection. These data are abridged in Table 2.

**Table 2:** Summary of key findings from TNF-related studies in cattle

Study (Year) Ref #	Sample number	Breed	Study design	PVL assay/threshold	Main endpoint	Effect direction & size	*p*-value/ Odds Ratio
Yudin et al. (2006) [7]	219	Various Russian breeds	Association (cross-sectional)	NA	*TNF-α* − 824 A > G vs. TNF + PBMC subsets	G/G genotype → higher TNF + PBMCs (CD11b+, IgM+)	*P* < 0.03
Konnai et al. (2006) [25]	291	Japanese Black & Holstein	Association (cross-sectional)	NA	*TNF-α* − 8*24* A > G vs. disease progression markers	G/G genotype → higher TNF + PBMCs; associated with progression (size NR)	*TNF-α* − 8*24* A > G *p* = 0.011
Bojarojc‑Nosowicz et al. (2015) [22]	1,200	Polish dairy cattle	Cross-sectional	**Low PVL**: < 10^3 copies × 10^5 PBMCs **Moderate PVL**: 10^3 to 10^5 copies × 10^5 PBMCs **High PVL**: 10^5 copies per 10^5 PBMCs	*TNF-α* − 8*24* A > G vs. mTNF-α protein and mRNA	G/G → ↑ mTNF-α protein despite ↓ mRNA (size NR)	*TNF-α* − 8*24* A > G *P* < 0.05
Stachura et al. (2016) [14]	120	Polish dairy cattle	Cross-sectional association	qPCR Low: < 10^3, Moderate: 10^3–10^5, High: >10^5 copies per 10^5 PBMCs	*TNF‑RII* rs42686850 & (CA)n vs. TNF + cell subsets	Microsatellite linked to TNF+, CD11b + TNF+, IgM + TNF + proportions	*p* < 0.05
Ohnuki et al. (2021) [23]	9 samples (1 FLK-BLV cell line, 4 EBL tumor samples, 4 non-EBL blood samples)	Cattle (BLV–Japanese)	Targeted DNA‑capture sequencing	DNA-capture-seq approach using next-generation sequencing	*TNF‑α − 824* G variant linked to BLV integration/clonal expansion	Association reported; effect size NA	NA
Mingala et al. (2009) [13]	445	Water buffalo (riverine vs. swamp)	Comparative expression study	qPCR NA	Cytokine expression (IL‑4, IL‑6, IL‑10, IL‑12p40, IFN‑γ)	↑ TNF‑α & IFN‑γ in swamp vs. riverine; BLV‑infected showed cytokine shifts	*P* < 0.05
Cheng et al. (2016) [24]	164	Cattle (Holstein; details NR)	Case–control (bovine TB susceptibility)	NA	*TNF‑α* promoter variants (incl. rs1800630) vs. TB susceptibility	Variant associated with *M. bovis* susceptibility (size NA)	*p* < 0.05

NA: Not Applicable

PVL: Proviral Load

### Genome-Wide association studies (GWAS) and novel loci identified

Advances in high-throughput genotyping and GWAS approaches have broadened the understanding of BLV resistance. This knowledge now extends beyond classical immune-related genes. Several studies have uncovered new loci associated with PVL and susceptibility to BLV infection. These findings indicate that resistance is a polygenic trait. Multiple pathways are involved, including antigen presentation, vesicular trafficking, lipid metabolism, transcriptional regulation, cytokine signaling, and the control of apoptosis.

#### SPATA16 (Spermatogenesis associated 16)

Mekata et al. (2022) identified SPATA16, a gene primarily known for its role in spermatogenesis and intracellular vesicular trafficking associated with the Golgi apparatus [26, 27]. Notably, in Japanese Black cattle, a SNP within SPATA16 was significantly associated with PVL, a marker of BLV PVL; animals carrying the minor allele exhibited approximately 20–25% lower median PVL than major-allele homozygotes [28]. Although the underlying mechanism remains uncertain, SPATA16 may influence BLV replication through effects on intracellular protein processing or vesicle-mediated transport. Consequently, this positions SPATA16 as a promising non-immune candidate marker for BLV resistance.

#### PNPLA1 (Patatin-Like phospholipase Domain-Containing protein 1)

PNPLA1 is a member of the patatin-like phospholipase family implicated in lipid metabolism and membrane remodeling [29, 30]. Notably, Brym et al. (2016) found SNPs near PNPLA1 that are associated with BLV infection status [31]. Since viruses often exploit host lipid pathways for replication and budding, polymorphisms in PNPLA1 may alter membrane composition, potentially affecting viral egress and persistence. Together, these findings suggest a mechanistic link between PNPLA1 variants and BLV infection.

#### AP4B1 (Adaptor related protein complex 4, beta 1 Subunit)

AP4B1 encodes a component of the adaptor protein complex 4 (AP-4). By regulating vesicle formation and intracellular protein sorting from the trans-Golgi network to endosomes [32], AP-4 plays a significant role in both cellular and viral protein trafficking. In the context of BLV infection, polymorphisms in AP4B1 may alter BLV assembly and release, as vesicular transport mechanisms are central to retroviral budding [31, 33]. Changes in AP-4 function within B lymphocytes, therefore, could affect the intracellular trafficking of BLV components and potentially impact disease processes.

#### TRIM45 (Tripartite motif containing 45)

TRIM45 belongs to the TRIM family of E3 ubiquitin ligases, key regulators of innate immune responses, antiviral defense, and cell proliferation [34, 35]. TRIM proteins can restrict viral replication by promoting the degradation of viral proteins or enhancing interferon signaling [36]. Therefore, genetic variation in TRIM45 may alter the efficiency of antiviral signaling, influencing host resistance or susceptibility to BLV infection.

#### CDCA2 (Cell division Cycle-Associated protein 2)

SNPs near CDCA2 have been associated with BLV infection and PVL levels [31]. Notably, CDCA2 encodes a nuclear protein involved in chromatin organization and mitotic progression by regulating phosphatase PP1-mediated dephosphorylation of chromatin substrates [37, 38]. Since BLV infection disrupts cell cycle control and increases B-cell proliferation, variants in CDCA2 may, as a result, alter the gene’s function and thereby influence susceptibility to BLV infection as well as the efficiency of viral integration and expansion of infected clones [39].

#### ABT1 (Activator of basal transcription 1)

ABT1 functions as a transcriptional regulator that facilitates assembly and stabilization of the preinitiation complex for RNA polymerase II [40, 41]. GWAS identified SNPs near ABT1 associated with BLV infection status [31]. Because BLV replication relies on the host transcriptional machinery, allelic variation in ABT1 may affect viral gene expression and PVL integration by modulating transcription factor recruitment and chromatin accessibility.

#### IER3 (Immediate early response 3)

IER3 is a stress-responsive gene that is rapidly induced by viral infection, oxidative stress, and pro-apoptotic stimuli [42]. Importantly, through MAPK/ERK signaling, it regulates cell survival and maintains a balance between apoptosis and immune activation. SNPs near IER3 have been associated with variation in PVL, indicating the extent of BLV infection in B cells [43]. Notably, while BLV promotes B-cell proliferation and transformation, IER3 variants that influence apoptosis may determine whether BLV-infected cells undergo programmed death or persist, thereby directly impacting disease progression.

#### PRRC2A (Proline-Rich Coiled-Coil 2 A)

PRRC2A encodes an RNA-binding protein that regulates mRNA splicing, transport, and stabilization [44]. Furthermore, GWAS findings suggest that PRRC2A is involved in BLV infection [43]. Since BLV exploits the host’s transcriptional and post-transcriptional machinery, polymorphisms in PRRC2A may alter the expression of cell cycle or immune-related genes, thereby affecting the proliferation of infected cells and viral persistence.

To provide a clear overview of the loci identified through GWAS and their functional significance, we have summarized the key candidate genes associated with BLV susceptibility and PVL in Table 3. These genes represent a diverse array of biological pathways beyond classical immune responses, including transcriptional regulation (*ABT1*), stress response and apoptosis (*IER3*), RNA processing (*PRRC2A*), vesicular transport (*SPATA16*,* AP4B1*), lipid metabolism (*PNPLA1*), immune regulation (*TRIM45*), and cell cycle control (*CDCA2*). Each of these loci may contribute to BLV pathogenesis through distinct mechanisms, such as influencing viral replication, cell survival, immune evasion, or oncogenic transformation. By organizing these findings into a concise table, we aim to highlight their potential as genetic markers for selective breeding programs and as starting points for functional validation studies.

**Table 3:** GWAS-Identified genes associated with BLV susceptibility and PVL

Gene	Full name	Primary function	Proposed role in BLV infection	References
*SPATA16*	*Spermatogenesis Associated 16*	Involved in spermatogenesis, intracellular vesicular trafficking, and Golgi organization	May affect viral assembly or budding by modulating vesicular transport pathways, influencing BLV replication and PVL	Mekata et al., 2022
*ABT1*	*Activator of Basal Transcription 1*	Regulates preinitiation complex assembly and RNA polymerase II-mediated transcription	Variants may alter transcription initiation, affecting viral gene expression and integration efficiency	Brym et al., 2016
*IER3*	*Immediate Early Response 3*	Early stress response gene involved in apoptosis regulation and MAPK/ERK signaling	May control survival of infected B-cells under stress; anti-apoptotic variants could favor persistence and leukemogenesis	Petersen, M. I. et al., 2021
*PRRC2A*	*Proline-Rich Coiled-Coil 2 A*	RNA-binding protein involved in mRNA splicing, transport, and stabilization	Could influence expression of cell cycle and immune-regulatory transcripts, supporting viral persistence and B-cell expansion	Petersen, M. I. et al., 2021
*PNPLA1*	*Patatin-Like Phospholipase Domain-Containing 1*	Lipid metabolism and membrane remodeling	May affect viral envelope formation and replication efficiency by altering lipid availability	Brym et al., 2016
*AP4B1*	*Adaptor Related Protein Complex 4 Beta 1 Subunit*	Mediates vesicle formation and protein sorting from Golgi to endosomes	Variants could impact intracellular transport of viral components, influencing BLV assembly and release	Brym et al., 2016
*TRIM45*	*Tripartite Motif Containing 45*	E3 ubiquitin ligase regulating immune responses and antiviral signaling	May modulate innate immunity and act as a restriction factor; polymorphisms could alter host antiviral defense	Brym et al., 2016
*CDCA2*	*Cell Division Cycle Associated 2*	Controls chromatin organization during mitosis and phosphatase regulation	Variants may influence cell cycle progression, viral integration, and clonal expansion of infected cells	Brym et al., 2016

### Cell cycle regulators and BLV-related oncogenesis

Although much research on BLV susceptibility has concentrated on immune-related genes such as *BoLA* and cytokine regulators, several studies have investigated individual genes outside these pathways. These genes play roles in cell cycle regulation, apoptosis, immune modulation, and intracellular transport, which are all processes relevant to viral persistence and tumorigenesis in BLV-infected cattle. Below, we review the key genes identified and discuss their biological significance.

One such gene is *Cyclin-Dependent Kinase Inhibitor 1 A; CDKN1A (WAF1/CIP1)*, a cyclin-dependent kinase inhibitor regulated by the p53 pathway, known for its role in cell cycle arrest and apoptosis [45, 46]. It encodes an inhibitor of cyclin-dependent kinases (CDKs), which control cell cycle progression from G1 to S phase. By inhibiting CDK activity, p21 enforces cell cycle arrest in response to DNA damage, thus preventing uncontrolled proliferation and allowing DNA repair or apoptosis [45, 47].

Because BLV is an oncogenic retrovirus that promotes B-cell proliferation, alterations in cell cycle regulators such as p21 could theoretically contribute to leukemogenesis. Dequiedt et al. analyzed bovine leukemic tissues from BLV-infected cattle for mutations in *WAF1/CIP1*. Surprisingly, no somatic mutations were detected, suggesting that p21 dysfunction is not a major driver of BLV-induced lymphoma [48]. This finding implies that BLV-induced oncogenesis likely bypasses the p53-p21 checkpoint through alternative mechanisms, such as Tax-mediated transcriptional dysregulation or epigenetic silencing of other tumor suppressors, rather than direct mutation of p21.

### Intrinsic restriction factors and BLV infection

In addition to classical immune-related loci and GWAS-identified genes, intrinsic restriction factors play an important role in controlling retroviral infections [49, 50]. These intrinsic antiviral proteins act at the cellular level to restrict viral replication, often at early stages of the viral life cycle. Although their role in BLV infection has not been comprehensively characterized, several key families are worth highlighting:

#### APOBEC3

Members of the APOBEC3 family are cytidine deaminases that introduce hypermutations into retroviral genomes, thereby impairing viral replication [50, 51]. While APOBEC3 proteins are well established as potent restriction factors in other retroviruses such as HIV, studies directly linking bovine APOBEC3 polymorphisms to BLV susceptibility are still lacking [50, 51]. Nevertheless, their conserved antiviral activity suggests a potential role in shaping BLV evolution and host-virus interactions.

#### BST2/Tetherin

BST2 prevents enveloped virions from leaving the host cell by anchoring them to the membrane, thereby restricting viral spread [52]. In cattle, Takeda et al. (2012) identified three isoforms of bovine BST2 (bBST2A1, bBST2A2, and bBST2B), each with distinct antiviral activity against bovine retroviruses [53]. Liang et al. (2017) later demonstrated that BST2A1 restricts both the release and cell-to-cell transmission of retroviruses, underscoring its role in bovine innate antiviral defense [54]. While direct studies on BST2’s role in BLV infection are few, these results suggest that tetherin likely restricts BLV virion release and spread among infected lymphocytes [55].

#### TRIM family

Tripartite motif-containing (TRIM) proteins are widely recognized as antiviral restriction factors that regulate innate immune signaling and directly inhibit viral replication [49]. TRIM5, for example, is a well-known retrovirus restriction factor, while *TRIM45* has been highlighted in GWAS as a locus associated with BLV PVL [31]. This suggests that members of the TRIM family may act as genetic determinants of resistance, linking intrinsic antiviral defense with host genetic variation.

Taken together, while direct SNP-based evidence for APOBEC3 and BST2 in BLV infection is still lacking, these restriction factors remain promising candidates for future studies. Their potential interplay with GWAS-identified loci such as *TRIM45* underscores the importance of integrating intrinsic antiviral mechanisms into the broader genetic framework of BLV resistance and susceptibility.

## Conclusion

The collective findings from these studies highlight the significant role of host genetic variation in determining susceptibility to BLV infection and disease progression. Polymorphisms in key immune-related genes particularly in the Major Histocompatibility Complex *(BoLA-DRB3*), *TNF-α*,* TNF-RII*, and cytokine regulatory elements are consistently associated with differences in PVL, immune response profiles, and resistance across breeds and even across species (e.g., buffalo subspecies).

In addition to classical immune loci, genome-wide association studies have identified novel contributors to BLV resistance, including *PRRC2A*,* AP4B1*,* TRIM45*,* SPATA16*,* ABT1*,* IER3*, and *PNPLA1*. These discoveries highlight that resistance is a polygenic trait influenced by multiple genetic pathways, including those that regulate cell proliferation, immune modulation, and apoptosis.

Beyond these adaptive and regulatory immune genes, congenital restriction factors such as APOBEC3, BST2/Tetherin, and TRIM family proteins also represent important candidates in BLV control. While direct evidence of SNP associations in *APOBEC3* and *BST2* with BLV outcomes is currently lacking, their established antiviral roles in other retroviral infections suggest they may contribute to shaping BLV replication and persistence. TRIM proteins, particularly TRIM45, already emerge as promising links between innate restriction mechanisms and GWAS-identified resistance loci.

The integration of high-throughput genotyping, SNP-based expression profiling, and target-enrichment sequencing technologies offers powerful tools for dissecting the host-virus interface. Future research should incorporate congenital restriction factors alongside classical immune genes to fully capture the spectrum of host determinants influencing BLV infection. Such insights will not only deepen our understanding of BLV pathogenesis but also open avenues for selective breeding, diagnostic biomarkers, and immunogenetic interventions aimed at reducing BLV prevalence in cattle populations.

## Data Availability

No datasets were generated or analysed during the current study.
